# F170. SCHIZOPHRENIA POLYGENIC RISK SCORE ASSOCIATED WITH LEFT TEMPORAL GYRIFICATION

**DOI:** 10.1093/schbul/sby017.701

**Published:** 2018-04-01

**Authors:** Igor Nenadic, Stephanie Gräger, Swapnil Awasthi, Kerstin Langbein, Maren Dietzek, Bianca Besteher, Stephan Ripka, Markus M Noethen, Heinrich Sauer, Christian Gaser, Franziska Degenhardt

**Affiliations:** 1 Philipps-Universität Marburg - UKGM; 2 Jena University Hospital; 3 MIT; 4 MIT / Charite; 5 Bonn University

## Abstract

**Background:**

Brain structural changes in schizophrenia are thought to arise in part from genetic liability, as shown in studies of twins and siblings. Polygenic risk scores (PGRS) derived from large-scale genome-wide association studies (GWAS) have allowed to use measures of genetic liability calculated from large numbers of individual single nucleotide polymorphisms (SNPs). Initial studies on PGRS and structural imaging have, however, failed to provide clear associations. We used three separate measures of brain morphometry (voxel-based morphometry, cortical thickness, and gyrification) in a sample of healthy subjects to associate them with PGRS for schizophrenia in order to test the hypothesis that gyrification, a putative indicator of early brain development.

**Methods:**

We analysed high-resolution MRI scans (3 Tesla, T1-weighted MPRAGE, 1x1x1mm resolution) from n=153 healthy subjects with not current or previous psychiatric condition recruited from the local community. DNA from each subject was analysed using the PsychChip, and polygenic risk scores were calculated for schizophrenia, as well as bipolar disorder and major depression (for assessment of relative specificity of the schizophrenia PGRS). MRI data were pre-processed with the CAT12 toolbox (dbm.neuro.uni-jena.de/cat12) for analysis using a) voxel-based morphometry (VBM), b) cortical thickness, and c) gyrification (calculated using the absolute mean curvature approach (Luders et al., NeuroImage 2006). We initially used p<0.001 uncorr. on the peak-level and performed correction for multiple-comparisons on the cluster level.

**Results:**

We found a negative correlation of the schizophrenia polygenic risk score with gyrification in the left anterior superior cortex (i.e. the higher risk score loading the lower local gyrification), which was significant at the cluster-level for FWE correction (p<0.047). There was not such significant finding for positive correlations, nor for any of the VBM or cortical thickness analyses. Also, there was not significant association (positive or negative) with major depression or bipolar disorder PGRS in any of the three morphometry analyses.

**Discussion:**

Our findings suggest that SNP-based genetic risk for schizophrenia is associated with left temporal gyrification, a putative indicator of early brain development, which again might be affected by multiple schizophrenia risk genes regulating cortical formation and connectivity. Furthermore, our findings are consistent with the notion of specificity for both morphometric marker (i.e. gyrification, but not VBM or cortical thickness) as well as diagnosis (with negative findings for major depressive and bipolar disorder risk scores). PGRS might impact on early developmental markers of brain structure (and possibly function), rather than overall liability-related variance.

